# Abrogating endocrine resistance by targeting ERα and PI3K in breast cancer

**DOI:** 10.3389/fonc.2012.00145

**Published:** 2012-10-16

**Authors:** Emily M. Fox, Carlos L. Arteaga, Todd W. Miller

**Affiliations:** ^1^Department of Medicine, Vanderbilt-Ingram Cancer Center, Vanderbilt UniversityNashville, TN, USA; ^2^Department of Cancer Biology, Vanderbilt-Ingram Cancer Center, Vanderbilt UniversityNashville, TN, USA; ^3^Breast Cancer Research Program, Vanderbilt-Ingram Cancer Center, Vanderbilt UniversityNashville, TN, USA; ^4^Department of Pharmacology and Toxicology, Norris Cotton Cancer Center, Geisel School of Medicine at DartmouthLebanon, NH, USA

**Keywords:** PI3K, breast cancer, antiestrogen, aromatase, fulvestrant, tamoxifen, estrogen receptor

## Abstract

Antiestrogen therapies targeting estrogen receptor α (ER) signaling are a mainstay for patients with ER+ breast cancer. While many cancers exhibit resistance to antiestrogen therapies, a large body of clinical and experimental evidence indicates that hyperactivation of the phosphatidylinositol 3-kinase (PI3K) pathway promotes antiestrogen resistance. In addition, continued ligand-independent ER signaling in the setting of estrogen deprivation may contribute to resistance to endocrine therapy. PI3K activates several proteins which promote cell cycle progression and survival. In ER+ breast cancer cells, PI3K promotes ligand-dependent and -independent ER transcriptional activity. Models of antiestrogen-resistant breast cancer often remain sensitive to estrogen stimulation and PI3K inhibition, suggesting that clinical trials with combinations of drugs targeting both the PI3K and ER pathways are warranted. Herein, we review recent findings on the roles of PI3K and ER in antiestrogen resistance, and clinical trials testing drug combinations which target both pathways. We also discuss the need for clinical investigation of ER downregulators in combination with PI3K inhibitors.

## INTRODUCTION

At least 75% of breast cancers express estrogen receptor α (ER) and/or progesterone receptor (PR), which are tumor biomarkers of estrogen dependence. Antiestrogen treatments for patients with ER+ or PR+ breast cancer inhibit ER by antagonizing estrogen ligand binding to ER (tamoxifen and other selective estrogen receptor modulators, SERMs), inhibiting dimerization and downregulating ER (fulvestrant and other selective estrogen receptor downregulators, SERDs), or blocking estrogen production (aromatase inhibitors, AIs; letrozole, anastrozole, exemestane). While such endocrine therapies have changed the natural history of ER+ breast cancer, many tumors exhibit *de novo* or acquired drug resistance (**Table 1**). The only clinically validated mechanism of resistance to endocrine therapy is overexpression or amplification of the *ERBB2* (HER2) protooncogene (Arpino et al., 2004; De Laurentiis et al., 2005; Ellis et al., 2006). However, only 10% of ER+ breast cancers exhibit HER2 overexpression, prompting the need for discovery of other mechanisms of antiestrogen resistance.

**Table 1:** Frequencies of breast cancer recurrence and resistance to anti-estrogen therapies in patients with ER+ breast cancer.

Population	Treatment	Effect	Trial/reference
		Follow-up: 5 years 10 years	
Early-stage	Adjuvant anastrozole × 5 years	Distant recurrence: 9.8% 19.7%	ATAC (Cuzick et al., 2010)
Post-menopausal	Adjuvant tamoxifen × 5 years	Distant recurrence: 12.5% 24%	
		Follow-up: 5 years 8 years	
Early-stage	Adjuvant letrozole × 5 years	Recurrence: 14.5% 23.6%	BIG 1-98 (Regan et al., 2011)
Post-menopausal	Adjuvant tamoxifen × 5 years	Recurrence: 18% 28%	
Advanced			
Post-menopausal	Anastrozole	No clinical benefit: 33%	FIRST (Robertson et al., 2009a)
No prior Tx	Fulvestrant (high-dose regimen)	No clinical benefit: 27.5%	
Advanced			
Post-menopausal	Exemestane	No clinical benefit: 68.5%	EFECT (Chia et al., 2008)
Progressed on AI	Fulvestrant (loading-dose regimen)	No clinical benefit: 67.8%	
		Median follow-up: 5.3 years	
Disease-free following	Letrozole	With disease: 2%	MA.17
5 years of adjuvant tamoxifen	Placebo	With disease: 4.9%	(Goss et al., 2008)
		Median follow-up: 2.5 years	
Disease-free following	Exemestane	With disease: 9%	NSABP B-33
5 years of adjuvant tamoxifen	Placebo	With disease: 11%	(Mamounas et al., 2008)

A large body of experimental and clinical evidence suggests that hyperactivation of the phosphatidylinositol 3-kinase (PI3K) pathway, the most frequently mutated pathway in breast cancer, promotes antiestrogen resistance. PI3K is commonly activated by growth factor receptor tyrosine kinases and G-protein-coupled receptors in breast cancer cells. The signaling cascades triggered by PI3K, including PDK1, AKT, and SGK among others, promote cell growth and survival. For detailed information, we refer the reader to a recently published, comprehensive review of this material (Miller et al., 2011a). Herein, we focus on updated findings, clinical testing of drug combinations targeting the ER and PI3K pathways, and the need to clinically address the potential for continued ER signaling in patients treated with endocrine therapies.

## RATIONALE FOR COMBINED TARGETING OF THE ER AND PI3K PATHWAYS

We and others identified a requirement for PI3K in the estrogen-independent growth of long-term estrogen-deprived (LTED) ER+ breast cancer cells, which mirror clinical resistance to AIs (Sabnis et al., 2007; Crowder et al., 2009; Miller et al., 2010). Proteomic profiling revealed amplification of PI3K signaling via the mTOR substrates p70S6 kinase and p85S6 kinase, and the PI3K effector AKT in ER+ human breast cancer cells adapted to hormone deprivation. Treatment with the ATP-competitive PI3K/mTOR dual inhibitor BEZ235 (Maira et al., 2008) completely suppressed the emergence of hormone-independent ER+ cells and induced apoptosis in cell lines harboring activating mutations in *PIK3CA* (gene that encodes the p110α subunit of PI3K) or PTEN loss (PTEN antagonizes PI3K signaling). In contrast, the TORC1 inhibitor everolimus (Schuler et al., 1997) had only a partial effect (Miller et al., 2010; Sanchez et al., 2011). This partial effect may be attributable to feedback activation of PI3K/AKT upon inhibition of TORC1 (O’Reilly et al., 2006; Carracedo et al., 2008; Miller et al., 2009), suggesting that direct inhibitors of PI3K may be more effective than rapalogs in this setting.

**FIGURE 1:**
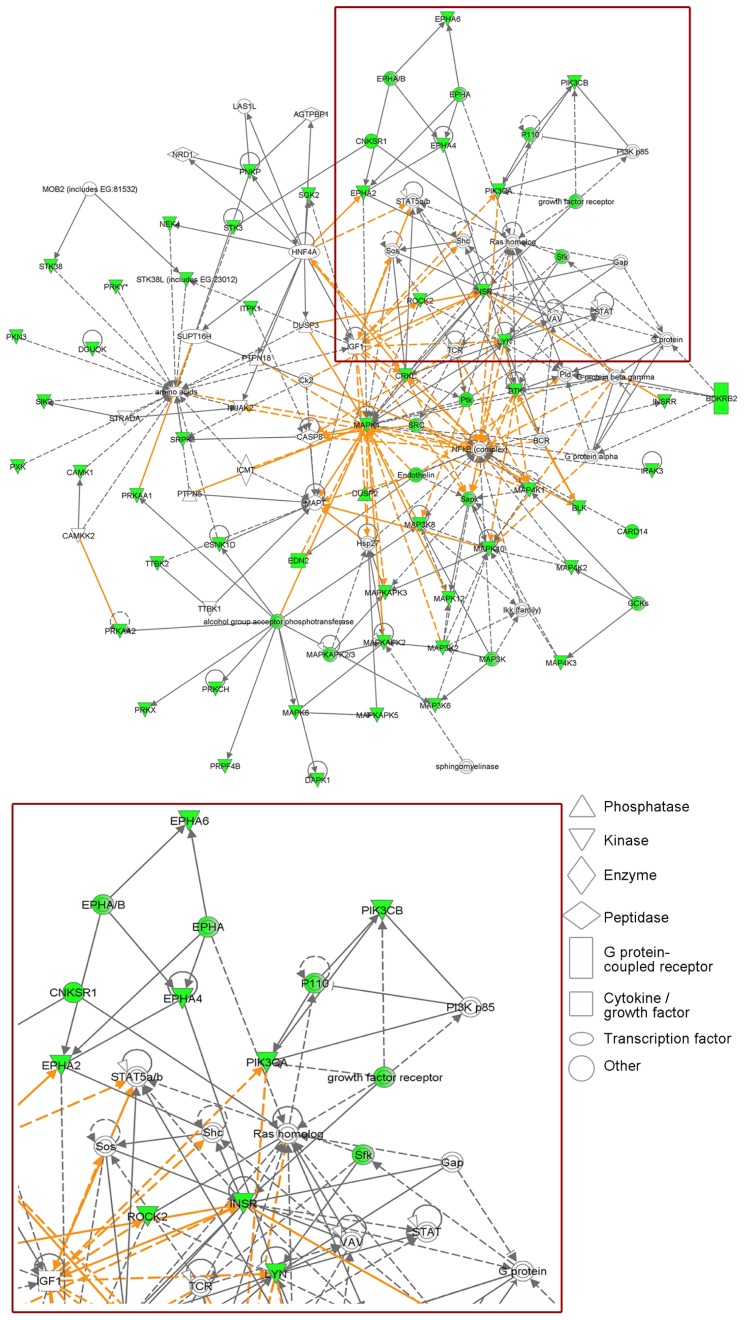
**Phosphatidylinositol 3-kinase is a central hub in signaling pathways required for estrogen-independent ER+ breast cancer cell growth.** MCF-7/LTED cells transiently transfected with a siRNA library targeting 779 kinases were reseeded in hormone-depleted medium. Cell viability was measured 4–5 days later by Alamar blue assay. Median cell growth in four independent experiments was calculated for each kinase siRNA relative to non-silencing controls. Individual knockdown of 42 kinases inhibited MCF-7/LTED cell growth ≥33% (*p* ≤ 0.05) in at least three of four experiments (detailed in Fox et al., 2011). Ingenuity Pathways Analysis revealed that these 42 kinases map to several protein networks that overlap with PI3K signaling (red box, enlarged in bottom panel). Proteins involved in these networks are displayed as nodes. Solid lines indicate direct relationships between proteins, and dotted lines indicate indirect interactions. Green nodes represent the kinases identified in the screen, as well as others whose knockdown was predicted by the Ingenuity software to negatively affect cell growth. The various nodal shapes represent the functional class of the gene product.

In a siRNA screen against 779 kinases, we implicated insulin receptor (InsR) in the hormone-independent growth of MCF-7/LTED cells. InsR and its homolog IGF-1R dimerize and, upon ligand binding, potently activate PI3K. IGF-1R has also been shown to confer antiestrogen resistance in MCF-7 cells (Zhang et al., 2011). Treatment with the ATP-competitive IGF-1R/InsR inhibitor OSI-906 suppressed PI3K activation and hormone-independent ER+ cell growth (Fox et al., 2011). Network mapping of the 42 kinases individually implicated in MCF-7/LTED cell growth in this screen revealed that PI3K is a central hub in these signaling pathways (**Figure 1**). Interestingly, a recent study showed that in ER+ breast cancer cells treated with BEZ235 or with PI3K siRNA, exogenous 17β-estradiol rescued the cells from drug- and siRNA-induced apoptosis (Crowder et al., 2009; Sanchez et al., 2011). This suggests that in ER+ cancers treated with PI3K inhibitors, estrogen suppression should be maintained and, therefore, combined inhibition of both PI3K and ER may be more effective than single-agent therapies.

**FIGURE 2:**
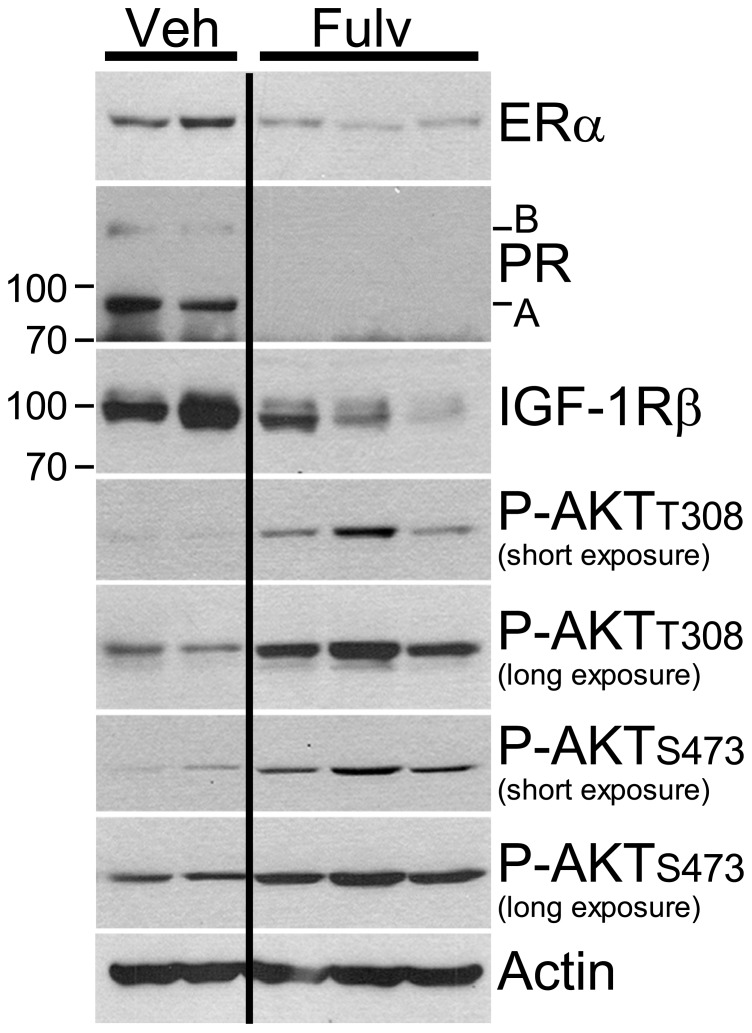
**Estrogen receptor inhibition with fulvestrant induces upregulation of PI3K signaling.** Ovariectomized athymic mice were s.c. implanted with MCF-7 cells and a 10-day-release E2 pellet (0.12 mg). Twelve days later, mice were randomized to treatment with vehicle or fulvestrant (5 mg/week, s.c., clinical formulation). Tumors were harvested after 3–4 weeks of treatment. Tumor lysates were analyzed by immunoblotting using the indicated antibodies; each lane contains equal amount of protein from two to three tumors. Fulvestrant treatment decreased the levels of ER and ER-regulated genes products (PR, IGF-1R), but increased levels of P-AKT-T308 and P-AKT-S473, suggesting increased activation of PI3K. All lanes were from the same membrane.

Clinical evidence further indicates that PI3K pathway activation is associated with antiestrogen resistance. Patients bearing primary ER+ breast tumors which exhibit a protein expression/phosphorylation signature of PI3K activation, as determined using reverse-phase protein arrays (RPPA), have a shorter recurrence-free survival (Miller et al., 2010). RPPA analysis of ER+ primary breast tumors obtained from patients following 2–3 weeks of treatment with the AI letrozole showed that a protein signature of insulin signaling was associated with high post-AI tumor cell proliferation (Fox et al., 2011). Overexpression of HER2 or FGFR1, or loss of INPP4B, molecular lesions which activate the PI3K pathway, also confer antiestrogen resistance in patients with ER+ breast cancer (Arpino et al., 2004; De Laurentiis et al., 2005; Ellis et al., 2006; Gewinner et al., 2009; Turner et al., 2010). Also noteworthy is the inverse correlation between levels of PI3K activation and ER protein in human tumors. This ER/PI3K balance can be shifted using PI3K and ER inhibitors in preclinical models (**Figure 2**; Creighton et al., 2010; Miller et al., 2010), suggesting that cells may defer to the other pathway when one is inhibited.

Crosstalk between the PI3K and ER pathways has also been suggested as a mechanism of endocrine resistance (Musgrove and Sutherland, 2009). PI3K activation was shown to induce ER phosphorylation at the putative AKT/p70S6K site Ser_167_ and estrogen-independent transcriptional activity (Campbell et al., 2001; Yamnik et al., 2009). However, treatment of such cells in hormone-depleted conditions with everolimus or the pan-PI3K inhibitor BKM120 (Maira et al., 2012) did not decrease ER phosphorylation at Ser_167_, ER-DNA binding, or ER transcriptional reporter activity (Miller et al., 2011b). These data collectively suggest that PI3K effectors do not modulate ER in the absence of estrogens. Analysis of the effects of BKM120 and fulvestrant on hormone-independent cell growth showed synergy in 6/8 ER+ lines. In mice bearing ER+ breast cancer xenografts, single-agent treatment with BKM120 or fulvestrant slowed tumor growth, while the combination induced tumor regression. Similarly, treatment with the ATP-competitive IGF-1R/InsR dual inhibitor OSI-906, which blocks downstream activation of PI3K in MCF-7 cells, slowed tumor growth and induced regression when combined with fulvestrant (Fox et al., 2011). These data further imply that combined targeting of the ER and PI3K pathways is more effective than single-agent therapies.

## CLINICAL TRIALS TESTING DRUG COMBINATIONS TARGETING THE ER AND PI3K PATHWAYS

Herein, we will review three recent clinical studies that evaluated the benefit of adding the TORC1 inhibitor everolimus to endocrine therapy. (1) In the first study, post-menopausal women with early-stage ER+ breast cancer were randomized to neoadjuvant therapy with the AI letrozole ± everolimus for 4 months. The addition of everolimus increased clinical response and suppression of tumor cell proliferation at 2 weeks (measured by Ki67 IHC) compared to letrozole alone (Baselga et al., 2009). (2) In the TAMRAD study, post-menopausal patients with metastatic, ER+, AI-resistant breast cancer were randomized to treatment with tamoxifen ± everolimus. The addition of everolimus improved clinical benefit rate, time-to-progression, and disease-free survival compared to tamoxifen alone (Bachelot et al., 2010). (3) The phase III BOLERO-2 study included 724 post-menopausal women with metastatic, ER+, HER2-negative breast cancer. While 84% of patients exhibited sensitivity to prior endocrine therapy, all were resistant to non-steroidal AIs (letrozole, anastrozole) at the time of randomization to treatment with the steroidal AI exemestane ± everolimus. The addition of everolimus increased progression-free survival (PFS) from 4.1 months (exemestane alone) to 10.6 months (Baselga et al., 2012).

While the addition of a TORC1 inhibitor prevents disease progression in patients with antiestrogen-resistant breast cancer, inhibition of TORC1 relieves negative feedback on activators of PI3K (e.g., IGF-1R, IRS-1, HER3; O’Reilly et al., 2006; Carracedo et al., 2008; Miller et al., 2009). These data suggest that direct inhibitors of PI3K may be more effective. Early clinical testing of PI3K inhibitors in combination with antiestrogens suggests that this strategy is feasible. In a phase Ib trial, post-menopausal patients with advanced ER+ disease are being treated with letrozole plus the PI3K inhibitor BKM120. This drug combination is safe and exhibits promising anti-tumor activity (Mayer et al., 2012).

## RATIONALE FOR AN ER DOWNREGULATOR IN COMBINATION WITH A PI3K INHIBITOR IN AI-RESISTANT BREAST CANCER

A recent comparison of high-dose fulvestrant (an ER downregulator) to the AI anastrozole as first-line treatment for advanced breast cancer revealed that fulvestrant provided a longer time-to-progression (Robertson et al., 2009a). In other studies, ~35% of patients who progressed on an AI responded to second-line fulvestrant (Ingle et al., 2006; Perey et al., 2007). This suggests that in some clinical situations, downregulation of ER may be superior to estrogen deprivation (AI) therapy (Robertson et al., 2009a). We recently reported that ER retains transcriptional activity in estrogen-independent LTED cells and primary human breast tumors (i.e., following AI therapy), and drives the estrogen-independent growth of LTED cells (Miller et al., 2011b). These data suggest that estrogen (ligand)-independent ER activity may promote resistance to AI therapy. While their side effect profiles are generally similar, AI therapy increases the risk of bone fractures and joint disorders more so than fulvestrant (Howell et al., 2002, 2005; Osborne et al., 2002; Goss et al., 2003; Robertson et al., 2003; Howell and Sapunar, 2011). Fulvestrant, which is administered intramuscularly, is associated with injection site pain, and only induces partial ER downregulation in tumors (Robertson et al., 2009b). Hence, the development of a more potent, orally available ER downregulator/inhibitor may provide a convenient and effective treatment option for patients with ER+ breast cancer.

**FIGURE 3:**
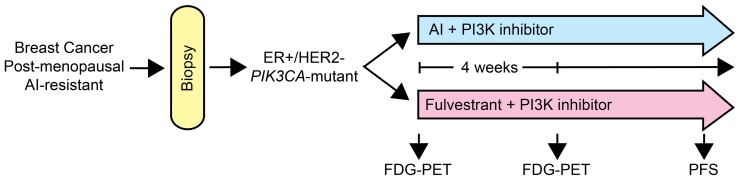
**Diagram of a clinical trial with a PI3K pathway inhibitor in AI-resistant breast cancer.** Patients with breast cancer that progressed on AI therapy will be subjected to a biopsy to confirm ER+, HER2-negative, *PIK3CA*-mutant status. Eligible patients would then be randomized to another AI plus a PI3K inhibitor, or fulvestrant plus the PI3K inhibitor. FDG-PET scans would be performed before and after 4 weeks of therapy to identify early metabolic changes. Patients will be treated until progression.

Cancer cells harboring activating mutations in *PIK3CA* exhibit increased sensitivity to PI3K inhibition (Miller et al., 2010; Sanchez et al., 2011; Maira et al., 2012), suggesting that this class of drugs may be most effective against tumors with mutations in the PI3K pathway. In mice bearing ER+, HER2-negative, *PIK3CA*-mutant MCF-7 breast cancer xenografts, treatment with the combination of fulvestrant and BKM120 induced tumor regression (Miller et al., 2011b). Using [^18^F]FDG-PET imaging as an early biomarker of metabolic inhibition, treatment with BKM120 but not fulvestrant decreased tumor FDG uptake. BKM120 increased tumor cell apoptosis, while fulvestrant decreased tumor cell proliferation. These findings may be validated clinically in a phase II clinical trial where post-menopausal patients with AI-resistant, ER+, HER2-negative, *PIK3CA*-mutant breast cancer are randomized to treatment with another AI plus a PI3K inhibitor vs. fulvestrant plus a PI3K inhibitor (**Figure 3**). The novel agent in such a trial would be the PI3K inhibitor, but the comparison would be an AI vs. fulvestrant. The primary endpoint would be PFS. Incorporation of non-invasive imaging with [^18^F]FDG-PET at baseline and after several weeks of treatment could identify metabolic changes indicative of a pharmacodynamic effect. This comparison would inform us whether (1) the addition of a PI3K inhibitor to an AI is beneficial, (2) downregulation of ER is superior to estrogen deprivation (AI) therapy in the context of PI3K inhibition, and (3) metabolic inhibition at an early time point as reflected by FDG-PET is predictive of PFS.

## Conflict of Interest Statement

The authors declare that the research was conducted in the absence of any commercial or financial relationships that could be construed as a potential conflict of interest.
